# Bargaining and gendered authority: a framework to understand household decision-making about childhood vaccines in the Philippines

**DOI:** 10.1136/bmjgh-2022-009781

**Published:** 2022-09-30

**Authors:** Jonas Wachinger, Mark Donald C Reñosa, Vivienne Endoma, Mila F Aligato, Jhoys Landicho-Guevarra, Jeniffer Landicho, Thea Andrea Bravo, Shannon A McMahon

**Affiliations:** 1 Heidelberg Institute of Global Health, Heidelberg University Hospital, Heidelberg, Germany; 2 Department of Epidemiology and Biostatistics, Research Institute for Tropical Medicine - Department of Health, Muntinlupa City, Philippines; 3 International Health, Johns Hopkins University Bloomberg School of Public Health, Baltimore, Maryland, USA

**Keywords:** Qualitative study, Vaccines, Public Health, Health education and promotion, Child health

## Abstract

**Introduction:**

Targeted vaccination promotion efforts aimed at building vaccine confidence require an in-depth understanding of how and by whom decisions about vaccinating children are made. While several studies have highlighted how parents interact with other stakeholders when discussing childhood vaccination, less is known about the way in which vaccination uptake is negotiated within households.

**Methods:**

We conducted 44 in-depth interviews with caregivers of children under five in the Philippines who had delayed or refused vaccination. Interviews were conducted between August 2020 and March 2021 and were audio-recorded, transcribed verbatim and translated into English. Notions of intra-household vaccination bargaining emerged early during systematic debriefings and were probed more pointedly throughout data collection.

**Results:**

Parents as well as paternal and maternal families proved to be dominant stakeholders in intra-household bargaining for childhood vaccination. Although bargaining among these stakeholders was based on engrained, gender-based power imbalances, disadvantaged stakeholders could draw on a range of interrelated sources of bargaining power to nevertheless shape decision-making. Sources of bargaining power included, in descending order of their relevance for vaccination, (1) physical presence at the household (at the time of vaccination decision-making), (2) interest in the topic of vaccination and conviction of one’s own position, (3) previous vaccination and caregiving experience, and (4) access to household resources (including finances). The degree to which each household member could draw on these sources of bargaining power varied considerably over time and across households.

**Conclusion:**

Our findings highlight how bargaining due to intra-household disagreement coins decisions regarding childhood vaccination. Considering the risks for public health associated with vaccine hesitancy globally, we advocate for acknowledging intra-household dynamics in research and practice, such as by purposefully targeting household members with decision-making capacity in vaccination promotion efforts, aligning promotion efforts with available bargaining capacity or further empowering those convinced of vaccination.

**WHAT IS ALREADY KNOWN ON THIS TOPIC**

- Vaccine hesitancy is among the biggest threats to global health, and the fallout of suboptimal vaccination rates is particularly acute in low- and middle-income countries.
- Decisions regarding childhood vaccination are commonly negotiated among caregivers; intra-household disagreements regarding a preferred course of action can spark conflict.
- Little is known regarding the intra-household decision-making process and the bargaining power available to household members.

**WHAT THIS STUDY ADDS**

- Based on qualitative data from the Philippines, we developed a framework outlining intra-household bargaining power when negotiating childhood vaccination.
- In the context of vaccination, gender-based power imbalances serve as an underlying structure for intra-household bargaining, but stakeholders can draw on a range of other sources of bargaining power to influence the decision.
- Physical presence at the time of vaccination decision is a key source of intra-household bargaining power, together with interest in the topic, access to household resources and previous caregiving and vaccination experience.
- These sources of bargaining power interact dynamically and can vary between households and over time.

**HOW THIS STUDY MIGHT AFFECT RESEARCH, PRACTICE OR POLICY**

- Researchers and implementers should consider the likelihood of intra-household disagreement and decision-making processes in their vaccine promotion efforts.
- Application of our framework in design and implementation work would facilitate: addressing actors with substantial bargaining power to consider vaccination, or empowering those actors already in favour of vaccination but currently possessing limited bargaining power to enact this preference.
- We encourage development and testing of approaches specifically designed to leverage bargaining-based opportunities, including, for example, efforts to encourage grandparents’ engagement for vaccination, to invoke fathers’ interest in childhood vaccines or to strengthen maternal agency based on their own caregiving experience.

## Introduction

Even before the onset of the COVID-19 pandemic, prominent researchers, policymakers and international bodies including the WHO repeatedly warned that surging vaccine hesitancy (VH) may upend decades of progress in improving child and adolescent health.^1–3^ Risks associated with expanding VH are especially acute in low- and middle-income countries (LMICs) where disease outbreaks place additional burden on already-strained health systems. Scholars have repeatedly argued that developing new and effective approaches to increase vaccine confidence requires a comprehensive understanding of factors influencing VH and the decision-making processes behind vaccination.^3–5^ Although literature outlining individual vaccination attitudes has expanded in recent years, less is known regarding how the decision to vaccinate is negotiated between individuals. This is particularly problematic in the context of routine childhood vaccination, where decisions commonly are not made by the vaccinated individual themselves, but by children’s caregivers (including a child’s parents, but potentially also other household members such as grandparents). In such contexts, conflicting opinions regarding vaccination can spark intrafamilial discussion and conflict.^6^

Evidence suggests that within households (for the purpose of this article defined as ‘a group of two or more persons living together who make common provision for food or other essentials for living’^7^), different household roles, rooted in religious, cultural and gendered norms, influence who has a say in deciding whether a child is vaccinated.^8–10^ Factors positively influencing vaccination uptake include the father’s educational background,^11^ the father being the one making the decision in favour of vaccination^11^ ^12^ and higher women’s empowerment.^13^ However, the influence of different household members can vary greatly across settings; pregnant mothers in Morocco reported a strong influence of their families on the vaccination decision,^14^ while immigrant mothers in Canada conveyed that their husbands played no substantive role.^15^ A recent meta-ethnography found that women’s lower social status routinely manifested as a barrier for vaccination uptake, including by limiting access to resources (eg, monetary resources required to address hidden costs of vaccination) and driving fears of negative consequences (eg, family conflicts in the event of negative vaccination side effects).^8^ Additionally, one systematic review suggested a potential role of grandparents in the vaccination decision, in part based on their first-hand experience with vaccine-preventable diseases, but highlighted a dearth of literature.^16^ The available evidence therefore indicates different household members with various types of agency and expertise potentially influencing the vaccination decision, but little is known regarding the nature of intra-household bargaining that underpins later vaccination decisions. One qualitative study investigated parental vaccination decision-making in the USA, but focused on the parent-dyad and how parents negotiated the decision with others and less on cases where parents themselves disagreed.^17^

Although evidence for the role of intra-household bargaining in the decision-making process for childhood vaccination is limited, substantial evidence from other fields of child health highlights the key importance of bargaining between household members. A study in Ethiopia,^18^ mirroring findings from other studies in western Africa,^19 20^ found that mothers commonly identify childhood illness, but have to bargain with other household members to seek care; mothers are commonly constrained in their bargaining power by structural and cultural factors. Constraints highlighted across studies as limiting women’s bargaining power for child health are a reduced agency to access or make decisions involving financial costs, as well as deeply gendered decision-making, which often favours men’s opinions over women’s.^10 18 21^ Financial agency is potentially less relevant in the context of childhood vaccines, which in many settings are available free of charge at health facilities (although incurring costs associated with transportation or loss of income). The evidence regarding extensive and often gendered intra-household bargaining for child health, however, merits attention to bargaining processes when decisions about childhood vaccination are being made.

Considering the urgent need for novel approaches for vaccine promotion efforts, understanding these bargaining processes could provide valuable insights for programme planners and policymakers, including regarding potential target actors or ways to empower household members in favour of vaccination. In this article, we present data from a qualitative study among vaccine hesitant caregivers in the Philippines to outline the power dynamics underpinning intra-household bargaining for childhood vaccination.

### Theoretical background

The outlined literature gap with regard to intra-household vaccination bargaining may be linked to methodological and economical challenges for collecting intra-household data,^22^ but also to historical, and from the end of the 20th century onwards heavily critiqued conceptualisations of households as unified, pooling cooperatives.^22–24^ Traditionally, scholarship related to general intra-household bargaining often focused on the influence of gender and gender roles on resource allocation, with a particular emphasis on male control over financial resources.^25^ More recent literature argues that access to resources should be seen as just one facet of power because even where women can access household finances, their say in how money is spent can be limited (due to, eg, sociocultural norms or intra-household restrictions).^21^ Scholars thus urge for intra-household bargaining research that reaches ‘beneath the surface of gender inequalities’ to more complex, interrelated dynamics.^21^

While several established frameworks conceptualise gender-based asymmetries,^26^ analytical starting points for examining intra-household asymmetries that extend beyond gender are limited.^22^ One such framework, the Intrahousehold Disadvantages Framework (IDF), proposes first identifying clusters of disadvantages within a given community, and then assessing via individual case-studies factors that underpin disadvantage including vulnerabilities and capabilities,^22^ acknowledging how all individuals possess capabilities (eg, goods, skills, networks, etc) but the balance between vulnerabilities and capabilities can vary.^27^ Moving beyond gender, the IDF also considers an individual’s relationship to the household head, the number and birth order of children in a household, disability and ill health, and age as variables that can underpin power.^22^ Based on these considerations, for this article we conceptualise intra-household bargaining as being coined by (1) several actors who have varying capabilities and vulnerabilities across (2) several interrelated dimensions of bargaining power within and beyond gender.

## Methods

### Study setting

This study is part of a larger project to co-develop vaccine promotive messaging and interventions in the Philippines.^28^ Across the country, routine vaccinations for children are available free of cost at government facilities as part of the national Expanded Program on Immunization.^29^ Although basic immunisation is formally mandatory in the Philippines for all infants under a 2011 Republic Act,^30^ institutionalised repercussions on a national scale for caregivers not vaccinating their children are limited. However, some elementary schools require proof of routine vaccination on enrolment, substituted by large-scale school-based immunisation campaigns,^31^ and low-income families aiming to obtain certain welfare benefits have to meet child vaccination and health check-up targets among other conditions.^32^

After a long period of high vaccine approval and vaccination rates, the Philippines experienced a highly publicised vaccine scare starting 2017.^33^ New data indicated that Sanofi’s dengue vaccine Dengvaxia, which had been rolled out at schools across the country for over a year, posed unknown risks for children without previous history of dengue infection.^34 35^ This, combined with a politicisation of the ensuing discourse by several institutions, resulted in a rapid erosion of trust in vaccines and vaccination uptake,^33 34^ leading to outbreaks of measles and polio in the country.^34 36^ Vaccine confidence showed signs of recovery in the years prior to the COVID-19 pandemic,^33^ but pandemic-associated disruptions and public discourses as well as viral (mis-)information sparked new concerns.^34^ Additionally, scholars have highlighted the particular role of social media in the Philippines in the context of (anti-)vaccination discourses, both during the Dengvaxia controversy and the COVID-19 pandemic.^37 38^ This combination of a highly publicised vaccine discourse (prior to the ongoing pandemic) and its fallout in a setting with traditionally high vaccine confidence, coupled with vocal debates of the topic outside traditional media and the health system, makes the Philippines a particularly suited case to study how conflicting interests, experiences and convictions inform vaccination decision-making within households.

Regarding the role of bargaining in intra-household decision-making processes in the Philippines, scholars have highlighted how Filipino households are often characterised by a comparatively large degree of egalitarianism between male and female household co-heads.^39 40^ While a majority of available literature focuses on the relationship between female bargaining power and household finances,^39 41 42^ the observed role of bargaining in intra-household decision-making is likely to extend also to other contexts, including to decisions regarding health and health careseeking for children.

### Data collection and analysis

We conducted in-depth interviews with caregivers of children whose vaccination records showed a delay or refusal of at least one routine childhood vaccine. Eligible participants were recruited with the help of local health workers. Interviews were conducted between August 2020 and March 2021 and focused on respondents’ vaccination experiences and narratives, as well as rationales for or against vaccination. Members of the research team with 2–15 years of experience conducting qualitative research were trained for 3 days on qualitative data collection and conducted interviews following a piloted and refined semi-structured guide. In the context of COVID-19-associated lockdowns, we conducted interviews online after respondents provided written and video-recorded informed consent; our experiences with conducting online in-depth interviews and the associated challenges and mitigation approaches are published elsewhere.^43^ Based on our experiences with online focus group discussions with a similar group of respondents which proved not to be feasible amidst a number of logistical challenges and data quality concerns,^44^ and as we aimed to elicit nuanced and in-depth experiences from individual respondents, we decided to focus on in-depth interviews for the purpose of this study.

Interviews were scheduled at a time of the respondent’s choosing, and respondents were asked to participate from a private place they felt comfortable in. Due to the online nature of the interview, interviewers could not always guarantee that no other individuals came into hearing distance for parts of the interviews. In cases where interviewers noticed disturbances by others, they let respondents decide to continue, halt or reschedule the interview. No respondent preferred to reschedule or voiced privacy concerns. In one case, a child’s father took over the interview after his wife had to leave to take care of their child. This change was initiated by the mother herself, and the father expressed eagerness to share his opinion about the topic, taking over in the interview where his wife had left off and providing responses to the latter half of the interview questions; both individuals provided informed consent and expressed no interest to reschedule and complete full interviews individually. All interviews were conducted in Filipino, audio-recorded, transcribed verbatim and translated into English.

In the context of routine systematic debriefings^45^ held throughout data collection, themes related to intra-household conflict and bargaining associated with the vaccination decision-making process emerged as highly salient. After observing saturation (defined as ‘the point in data collection and analysis when new information produces little or no change to the codebook’^46^) in the context of these debriefings for themes related to intrahousehold bargaining across stakeholders, the lead author inductively refined the codebook based on debriefing notes and the coding of five purposefully selected information-rich transcripts. The codebook was adapted based on discussions within the team and ultimately applied iteratively to the entire dataset using NVivo V.12 (12.6.0., QSR International) following the tenets of framework analysis.^47^ Over the course of coding, sources of vaccine-related decision-making power emerged, and codes were integrated into a working framework based on the data and engagement with the literature.

Further information regarding the study setting and procedures are published elsewhere.^28^ A full COREQ checklist (online supplemental file S1) and an author reflexivity statement^48^ (online supplemental file S2) are included as supplemental files.

10.1136/bmjgh-2022-009781.supp1 Supplementary data

10.1136/bmjgh-2022-009781.supp2 Supplementary data

### Patient and public involvement

Patients and the public were not directly involved in the design of the study. However, following the tenets of human-centred design (for the overarching study) and qualitative research, the research team consistently collected participant narratives and feedback, and the results presented here give voice to these participant experiences.

## Results

We conducted 44 interviews with n=45 individuals (including the case of a father taking over for his wife described above). Interviews on average lasted 63 minutes (range 30–105 minutes). Most respondents were mothers (n=39), but primary caregivers interviewed also included fathers (n=3) and grandmothers (n=3). Respondents had an average age of 35 years (range 19–70 years) and between 1 and 11 children. Thirty-two respondents had at least high school education, with two respondents indicating that they had received no formal education.

**Figure 1:**
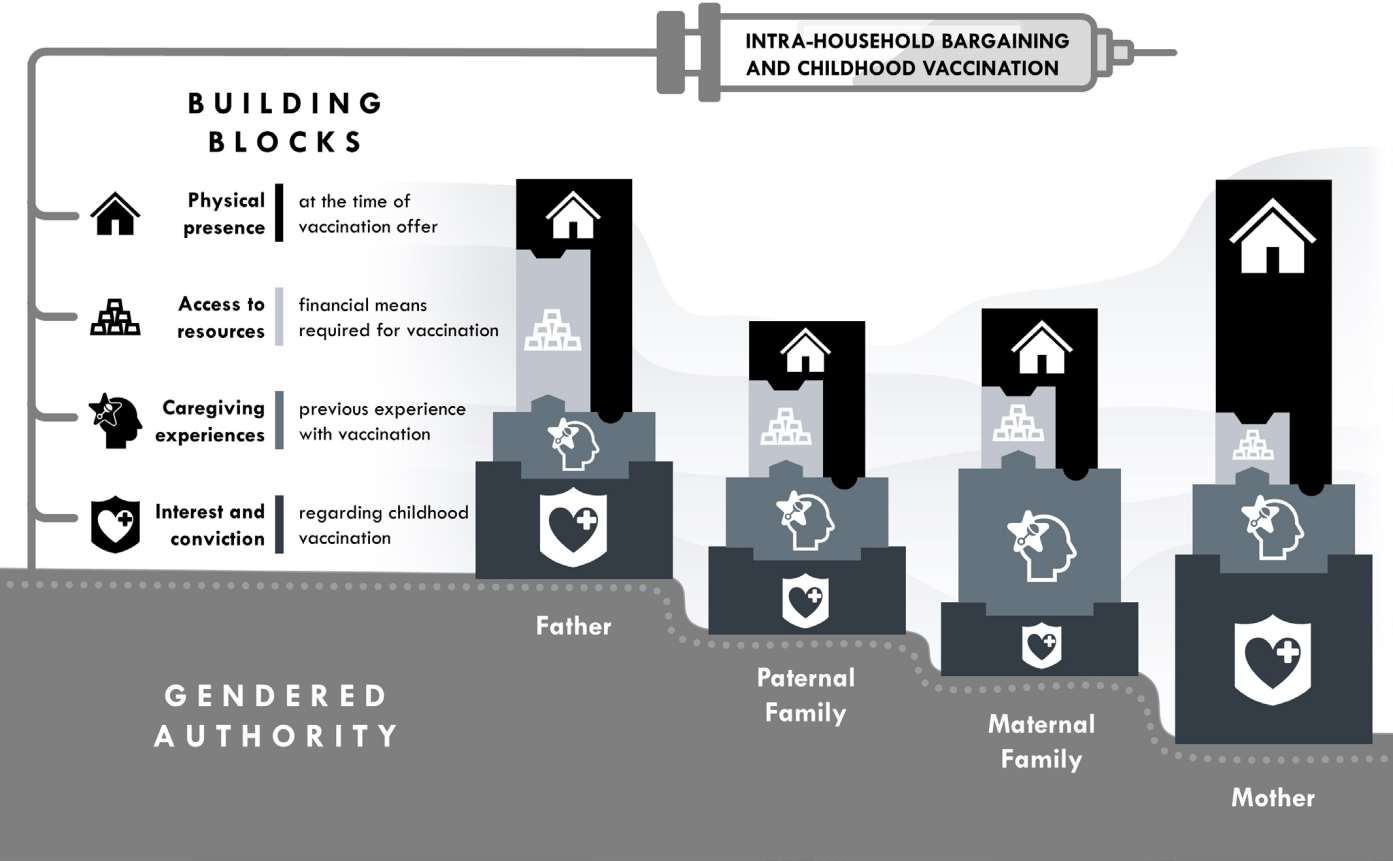
The bargaining and gendered authority framework for childhood vaccination.

Gendered authority and power formed an unequal basis on which bargaining took place among household members such as fathers and mothers, but also maternal and paternal extended families (with grandparents being the most influential actors in the bargaining process besides the child’s parents). Several sources of bargaining power emerged, which actors drew on when bargaining within households. Figure 1 presents the resulting bargaining and gendered authority framework.

### Structure of the framework

The framework conceptualises gender-associated imbalances in bargaining power as an underlying baseline, imbuing key actors with power and authority to make vaccine-related decisions. However, the building blocks of bargaining and decision-making can reshape or refute gendered power determinants: (1) being physically present at home when a decision is made; (2) having access to resources, especially monetary means; (3) possessing caregiving experience, especially with regard to childhood vaccination; and (4) having inherent interest in the topic of vaccination and conviction in one’s position (eg, vaccination being necessary or harmful). In the figure, the height of blocks represents a general tendency of power distribution within households as emerging from our respondents’ narratives (with, eg, fathers often drawing on their access to resources, while mothers described their presence in the household and their higher perceived caregiving experience). The actual distribution of different types of bargaining power in individual households or in other contexts can vary.

Based on the emphasis respondents placed on the different types of bargaining power in their narratives, we arranged the building blocks based on (1) their stability over time and (2) their relevance in the vaccination context. Stability over time was assessed based on instances emerging from respondents’ accounts of the different types of power being available continuously (or very stable or slowly increasing in nature), or only in certain instances (with a considerable likelihood of a sudden loss in power). This stability is represented in figure 1 via the width and vertical order of blocks (with physical presence changing most frequently and therefore being an instable source of power, while interest in the topic of vaccination was much more stable). Relevance of the respective source of power in the vaccination context was determined based on respondents’ narratives of what they perceived as being the factors frequently influencing household bargaining decisively versus factors influencing the decision only in certain situations. This relevance is visualised via the shading of blocks, with the more relevant sources of power being shaded darker: While, for example, physical presence emerged as highly relevant in the context of vaccines (shaded darker), the opposite is the case for access to resources (shaded lighter).

We begin by outlining the data on gendered authority as a latent source of power underlying household bargaining. We then present power domains based on their relevance in the vaccination context in descending order, followed by how these different sources of power can interact and change over time.

### Gendered authority and power

Gendered authority emerged as a latent source of power individuals could rely on when negotiating a decision. Mothers often emerged as having less authority than other household members, while fathers were, almost universally, the clear figure of authority in households. As one mother described it: “My husband feels that he should be superior [in the family] … He has more power than me” (mother of 2, 25 years, urban).

As a result, participating mothers reported that they were afraid to be held accountable for any negative consequences if they decided to go against their partner’s vaccination decision: “I already know that he does not like vaccinations. So, there was one time when I asked for his permission to go to the health center [with the child]. He said, ‘bahala ka sa buhay mo!’ [lit.: ‘It’s your life. Do what you want!’]. Those words, I already knew, it would mean a scary fight. So, I just stopped” (mother of 4, 24 years, urban). Similarly, one mother described how, despite her desire to vaccinate her child, she followed her husband’s opposition to vaccines “because I don’t want any trouble” (mother of 3, 34 years, urban). In cases where mothers previously had vaccinated their children but fathers later found out or changed their opinion, fathers’ authority allowed them to inhibit future vaccination: “He said that the children are already okay, … so he said that he would not allow the children to be injected again” (mother of 3, 34 years, urban).

Maternal grandparents often emerged as having authority over their daughters, but sometimes lacked influence over their sons-in-law. One mother narrated how she had turned to her own parents for help to convince her vaccine hesitant partner but “my husband doesn’t allow injections. My mother insisted and told him that it is for our child’s health. Still, he said no” (mother of 2, 25 years, urban). However, in one case a respondent also reported how her husband was “afraid of my father [the child’s grandfather]” who was opposing vaccination so “he did not say anything”, resulting in the child not being vaccinated as long as the family was living with the grandparents (mother of 5, 28 years, rural).

Paternal grandparents often had considerably more influence on the father than maternal grandparents. Additionally, mothers often feared potential consequences if they behaved against the will of their parents-in-law: “Every time I bring up the idea of vaccination, my mother-in-law says, ‘Go ahead, it is up to you! Inject your child, but when something happens, don’t ask for any help from me.’ They always say that I will be on my own, just because of the vaccine” (mother of 4, 24 years, urban).

### Physical presence

A key source of power influencing household bargaining was physical presence at the time of vaccination offer. Mothers emerged as often being the caregiver physically present at the household, including in situations when decisions about vaccination had to be made (eg, vaccination schedules, house-to-house campaigns), providing them with considerable decision-making power. As one mother described it when asked about her husband’s opinion on her vaccinating the children: “Nothing, Ma’am, he was at work… I told him only when he arrived” (mother of 7, 46 years, rural).

Consistent physical presence at home allowed some respondents to directly overrule preferences of other actors by deciding covertly. One respondent said that if her husband was present, their children “can’t be injected because he will get angry”, but “when he leaves then it’s time for me to go to the health center, so he doesn’t know” (mother of 3, 34 years, urban). Other respondents reported that they “hid the truth from him” because “my partner looks like he doesn’t want it” (mother of 2, 21 years, urban). However, such vaccination secrecy, once discovered, would often spark conflict: “He’s not around that time, but when we got home, he questioned me why I allowed my child to be vaccinated without his knowledge” (mother of 2, 25 years, urban).

Respondents also relayed how deriving one’s decision-making power from physical presence could be fleeting or heavily dependent on one’s own employment situation. Similarly, being the only one physically present might give an individual considerable decision-making power, but at the same time make it “hard because I’m alone here at home… It’s not like when you have someone to leave the other kids with” (mother of 2, 31 years, urban).

Mothers often described how fathers were typically working, oftentimes in distant regions, resulting in considerably diminished paternal decision-making power. One recently widowed participating father described only beginning to take care of his child’s medical needs after his wife’s death as previously “I don’t know what specific vaccine my child still needs, because my wife was the only one who held the record. I just work” (father of 3, 46 years, urban).

The role of physical presence as a source of power, although instable, was particularly present in the role of grandparents or extended family. Several respondents reported that maternal or paternal grandparents heavily influenced vaccination decisions at times when they were living nearby or were serving as primary caregivers to children: “this [grandchild] really lived with her mother before. Once we are able to travel again, I will return [him] to his mother, but I want to complete his vaccination first because [his mother] won’t take care of this” (grandmother of 2, 57 years, urban).

Mothers also explained an inability to continue vaccination if they lived with grandparents who opposed vaccination: “That is why when I was separated from [the children’s grandparents], then I had my children immunized” (mother of 11, 48 years, rural). One mother narrated how she struggled for years to vaccinate her children against the will of her own father and was only able to start vaccination once her father became unwell. With high physical presence, grandparents were also able to intercede on behalf of a parent, casting a tie-breaking vote for or against vaccination; this level of support often diminished after marriage, particularly for maternal grandparents when a mother moved away to live in the paternal household.

The role of physical presence was also exemplified in cases where families were temporarily divided due to the high mobility requirements for purposes of employment, schooling or taking care of family members. Such splits could result in different vaccination profiles among children of the same family, depending on whom they were living with during early childhood.

### Interest and conviction

Many respondents reported that they saw vaccination as a topic where household negotiations were heavily shaped by the person(s) taking interest in or holding strong convictions on the topic—whether for or against vaccination. Female respondents frequently described caring about vaccination as being part of their role as a mother and their own involvement in the topic as high, phrasing it as “I’m the parent, I’m the one who takes care of them, that’s how I am” (mother of 2, 21 years, urban). At the same time, some mothers also said their main reason for forgoing decisions about vaccination was being “too lazy to go” (mother of 5, 31 years, rural) or not seeing vaccines “as a priority” (mother of 4, 38 years, urban).

Some mothers described their husbands as not having “a care” (mother of 4, 38 years, urban) or that they “never had an opinion about the child’s health” (mother of 1, 23 years, rural), resulting in the second respondent feeling left alone with her worries when the child developed a fever after vaccination. However, one participating father described how vaccination was so important to him that even “if it will take the whole day, why not? If that is the only way to get [the vaccine], isn’t it? … Even if I lose my work, [it’s worth it] as long as my children will not get sick” (father of 3, 33 years, rural). Another father described feeling conflicted about vaccination at schools due to the Dengvaxia controversy: “I thought then if I allow my children to be vaccinated, I will not be able to sleep” (father of 3, 46 years, urban). He accordingly made sure that his children were fully vaccinated at a trusted health center, so that they could reject school-based vaccination.

Maternal grandparents often described their high interest (negative or positive) in the topic of vaccination as part of their duty to care for their grandchild’s health and to pass knowledge to their daughters: “I am not a type of mother who doesn’t care about her children. I made sure that all of them were seen by doctors and got the necessary care. [This way] they saw that [vaccination] is important to me, so they imitate it with their [own] kids. Because that’s who I am, I am their mother” (grandmother of 1, 50 years, urban).

Paternal grandparents mainly exerted power when caring about the topic of vaccines due to previous (negative) vaccination experiences, such as severe vaccination side effects in previously vaccinated children. One mother described how her mother-in-law was “the biggest barrier” because “she really doesn’t want her grandchildren to be vaccinated” (mother of 2, 21 years, urban). However, one paternal grandmother also explained how she was struggling as she herself cared about vaccines, while her former daughter-in-law did not. This resulted in the grandmother being concerned for her grandchild’s health as “when he was one year old, I gave him to his mother because my son and his mother separated… Yes, that’s why he was late [with the vaccine schedule], because his mother didn’t take care” (grandmother of 2, 52 years, urban).

### Caregiving and vaccination experience

Especially young mothers described how they saw other, typically older family members with more experience as having more power (and qualification) to decide about vaccination. Their own power increased with the amount of caregiving experience they possessed: While one mother described being “scared and nervous because that’s my first baby” (mother of 2, 29 years, urban), another mother said: “I already have experience because I have three children, it seems like I already know what is really being injected” (mother of 3, 34 years, rural). Mothers also frequently invoked their own experiences when explaining their hesitancies towards vaccines: “that’s why sometimes I am afraid to bring them to the health center because I am afraid that they would be hurt again” (mother of 6, 33 years, rural).

Fathers similarly invoked previous experiences with vaccination and vaccine side effects. However, some mothers described how fathers lacked experience with vaccines, also from their own childhood, as they were not common or “not really hindi uso [lit.: a trend]” (mother of 2, 25 years, urban) in their respective province, making fathers appear unsure about vaccine effectiveness and safety.

Participants explained how both paternal and maternal extended families held considerable power due to extensive caregiving and vaccination experience, describing them as “mas nakakaalam [lit.: those who are knowledgeable]” (mother of 3, 30 years, rural). One mother stopped vaccination after her father “got mad” because the child developed a fever after vaccination, and “since my father knew better than me, I followed him” in opposing vaccination (mother of 2, 29 years, urban). Another respondent explained how grandparents’ preferences were hard to overcome, and “even though I have 4 children, I still don’t know what is right for the children”, so “we’re just following [the grandparents’] advice for the children’s sake” (mother of 4, 24 years, urban).

### Access to resources

Control over resources was often distributed unevenly between household members, but generally did not emerge as a substantive source of bargaining power with regard to vaccination. Mothers reported how vaccines were generally accessible free of charge, unless one wanted to procure them at a private facility, but transportation to the centre could incur costs. However, participants highlighted the challenges if vaccines were to cost money, both hypothetically because they would have to then “ask my husband if he agrees” (mother of 2, 25 years, urban) or because they had been vaccinating their child at a private health facility but then “ran out of budget and then the injections were delayed” (father of 3, 46 years, urban).

### Interrelations between sources of power and dynamics over time

Sources of bargaining power emerged not in isolation from each other, but as mutually influencing, exacerbating or challenging. For example, physical presence could influence interest in the topic and one’s own caregiving experience. Similarly, having personal experiences with vaccination (both positive and negative) not only gave household members bargaining power, but also often increased the degree to which these members cared about the topic.

Household members who lacked high levels of gendered authority, especially mothers, were often overruled by fathers or extended family but could draw on combinations of other sources of power to mould the decision-making process. The degree to which this was possible could vary considerably over the course of their life. *Case study 1* highlights how changing circumstances within a mother’s life influenced the power she exerted in intrahousehold vaccination decision-making. Names of case-study respondents are pseudonymised.

**Case Study 1: Mary Joy**

Mary Joy, a mother of two, agreed to vaccinate her first child against polio. However, the child developed a mild fever and “was crying all night.” This disturbed Mary Joy’s father who was living in the same house and, consequently, opposed further vaccination. With the father being physically present, having previous childcare experience, and Mary Joy’s own conviction regarding the necessity of vaccines being low, the father ultimately enforced his decision.

Later, Mary Joy “realized how important [vaccination] is”, being convinced by other mothers and perceiving her unvaccinated child as being more prone to sickness than other children. However, despite frequent visits to the doctor, no further vaccination followed “because that time my mother is his guardian because I’m at work.”

The situation changed with Mary Joy’s second child, whose father lives apart from the family. Being the sole parent physically present and having gained caregiving experience, her second child is vaccinated following the recommended basic schedules—although she remains deeply skeptical towards newly introduced vaccines.

In addition to this variation over time, bargaining outcomes could also vary within the same household depending on the interlinked sources of power available to the respective household members. *Case study* 2 exemplifies a case where household context largely overlapped between two respondents who wanted to vaccinate their children, but their respective relationships with the other household members, previous experiences and bargaining power resulted in different outcomes.

**Case Study 2: Stephanie and Althea**

Stephanie, a married mother of three, lives in the same household as her parents, her brother and her sister-in-law Althea, a married mother of two. Both Stephanie and Althea would like to vaccinate their children, but while sharing one household, their access to power differs and shapes vaccination outcomes.

One of Stephanie’s children died when he was two years old. None of her children are vaccinated, as both her mother and her mother-in-law strictly oppose vaccines. Stephanie’s husband is similarly hesitant, but she imagines being potentially able to negotiate with him, but with both her mother and mother-in-law against vaccination, she feels isolated and fears potential repercussions. Once, she interacted with the health centre to get medicines for a sick child, and the facility staff “also urged us to take the children for vaccination. When I told my mother-in-law about the vaccines needed, she was so angry, she said: ‘Don’t take them to the health center, just take medicine. There is no need for the children to be vaccinated.’’’ Stephanie feels powerless and afraid as she cares about vaccines, and not being able to vaccinate her children “hurts me because I also want my children to be away from the pain, so they don’t get any diseases.”

Stephanie’s sister-in-law, Althea, faces similar opposition from the family matriarch, especially after having moved into the same household. Althea’s husband aligns with his mother in her vaccine hesitancy, resulting in Althea’s 8-month-old daughter not yet being vaccinated. However, Althea’s own parents advocate for vaccines, allowing Althea in the past to defy her husband and vaccinate their oldest son: “I was only with my mother and father. They knew about it, only my husband didn’t.” After returning to her husband, she “just told him what I did and it’s fine with him.” Drawing on these experiences, Althea intends to resist her mother-in-law, because “even if [the grandparents of the child] will not allow it, I will still let my children get vaccinated because their body needs it.”

Both participants, Stephanie and Althea, are convinced of the necessity of vaccinations, would like to vaccinate their children, but live in a household where their respective husbands and members of the extended family oppose vaccines. However, their own vaccination experiences and having the support of at least one side of the family have resulted in contrasting vaccination outcomes.

## Discussion

This study is among the first to outline sources of decision-making power that allow Filipino caregivers of small children to make decisions about vaccination in cases of intra-household disagreement. While gendered power (primarily available to fathers) emerged as an underlying foundation of intra-household imbalances, other household members could make and enact decisions based on (1) their physical presence at the household at the time when vaccination decisions are made or enacted, (2) high interest in the topic of vaccination, (3) previous caregiving or vaccination experiences and (4) access to household resources.

The role of gender underlying health-related household decision-making in our data is well established in the literature, both in relation to vaccination^8^ and child health in general.^21^ Our finding that different household members—beyond parents of small children—have considerable bargaining power in decisions regarding child health, and how gender dynamics shape the distribution of power, reflects previous scholarship. Beyond differences between genders, authors have also detailed cases of positive and negative influences of senior women, particularly mothers-in-law, on younger mothers.^21^ In Kenya, senior co-wives have been described as possessing extensive decision-making capabilities when engaging in (or rejecting) child health interventions.^49^ One additional factor that has been highlighted as potentially influencing such gendered power dynamics is (formal) education. While not emerging as a core determinant of gendered power from our data, strong evidence suggests that maternal education can increase women’s autonomy in the household and has positive effects on child health.^21^ In addition to reshaping the gendered power imbalances within households, in the context of our framework, education specifically about the relevance of vaccines could also influence the degree to which individuals care about the topic (although invoking such care might not automatically suffice to convince other household members).

The degree to which physical presence emerged as one of the key sources of power in vaccination decision-making was beyond what previous scholarship might suggest: While acknowledging its relevance, physical presence has mainly been conceptualised as one of several resources.^27^ In our work, the decision for vaccination can be made and enacted in one individual situation (eg, in door-to-door vaccination campaigns), and access to this source of power therefore can be much more fluid (and less finite when drawn on) than access to traditional resources such as household finances. The person physically present therefore can become a ‘de facto’ household head, able to make a decision in this particular moment (as opposed to the ‘de jure’ household head who might not be present).^21^ As highlighted in our results, this can also allow household members to decide and enact their decision secretly. While thereby ensuring their own preferred outcome, the discovery of such secrecy by other household members could spark conflict, and longer-term consequences remain unclear. Scholars have highlighted how having to keep secrets from others might increase perceived isolation and fatigue,^50^ and can severely impact family dynamics and result in an erosion of trust (between spouses, but also between children and one of their parents if instructed by the other to keep a secret), especially if found out.^51 52^ In the context of vaccination secrecy, later discovery or disclosure could therefore not only result in conflicts but also make future vaccination less likely due to the loss of trust between household members. At the same time, Wilson^53^ argues that, based on Foucault’s conceptualisation of secrecy being an integral part of power,^54^ household members might prefer to accept secrecy or feign ignorance as to not challenge the dominant ideology. Applied to our findings, household members could prefer to overlook vaccination secrecy as a means to maintain an appearance of sharing equal responsibility or decision-making power regarding their child’s health. We encourage future research to consider the effects of vaccination secrecy on household dynamics and future vaccination uptake.

Respondents repeatedly emphasised that caregiving experience gave stakeholders, particularly older family members, considerable authority, which confirms findings from other settings. In Senegal, grandmothers’ experience and wisdom gave them a role in maternal and child health, among others.^55^ A recent systematic review also highlighted that grandparents’ previous experiences, including of vaccine preventable illnesses, potentially allowed them to influence vaccination decisions but that in-depth research on the topic is currently lacking.^16^ Our study adds to this discourse by emphasising how previous caregiving experience can be a source of considerable bargaining power, meriting attention on senior household members in vaccination promotion efforts. At the same time, while having more than one child emerged from our data as an important determinant for confidence in one’s own caregiving experience for subsequent children, total number of children might not be the only metric relevant for such confidence. A study among Spanish mothers highlighted the relevance of not being primiparous, but also the number of people at home that could provide support for mothers’ caregiving confidence.^56^ Similarly, self-efficacy of mothers in the USA has been shown to increase both with maternal age and the number of children.^57^ These findings suggest a spectrum of factors influencing confidence in household member’s caregiving experience, although we would caution against broad comparisons given the high probability of contextual differences between these settings and our study site.

Childhood vaccination proved to be a sensitive topic among many caregivers, with those heavily invested in the topic often being key stakeholders in household bargaining. In line with our findings, conviction has been highlighted in the context of parental decision-making for children suffering from cancer where parents see it as their responsibility to decide what is right for their children;^58^ conflicts can emerge where these convictions are challenged by another party who also cares strongly about the topic but holds an opposing view (eg, medical providers).^59^ Vaccination programme planners should consider this high relevance of caring about the topic, for example, by invoking this care among those favouring vaccination in the household, but also by being aware of the bargaining power of stakeholders heavily opposed to vaccination.

The emergence of potential sources of bargaining power that interact with (and are co-dependent on) each other links to ongoing intersectionality discourses. Intersectionality highlights how inequalities associated with individual social stratifiers (eg, gender, class, age) do not exist in isolation from each other but instead interact dynamically.^60–62^ Amid calls for a more prominent role of intersectionality research in global health,^60 61^ scholarship that touches on intersectionality and vaccines predominantly focuses on HPV^63 64^ or COVID-19^65^ vaccines and highlights the role of intersectionality in all aspects of the vaccine delivery continuum. One principle frequently emphasised in intersectionality research is leveraging, describing how groups who have a combination of advantages and disadvantages can leverage their advantages to secure entitlements^66 67^—a finding reflected in our data related to vaccine decision-making. However, while our findings shed light on how emerging sources of bargaining power were not merely additive but influenced each other, further research specifically focused on exploring intersectional facets of childhood vaccination bargaining would facilitate further framework refinement.

Our study also reflects the previously highlighted prominent role of women in Filipino households,^39 40^ including notions regarding the relevance of a mother’s ‘own’ set of relatives she could rely on for support^68^ and how age can equalise, if not supersede, gender as a source of authority.^69^ The relevance of previous childcare in our data similarly resonates with findings regarding Filipino women’s authority generally increasing with the number of children.^70^ At the same time, scholars have argued that Filipino households might be more egalitarian than in many other countries, but that in cases of conflict the male preference nevertheless often prevails,^40 69^ mirroring our findings regarding fathers’ gendered authority. There are several marked differences between bargaining for childhood vaccination and household spending, including the diminished relevance of the economic dimension in the context of vaccination and childcare being predominantly understood as the mother’s responsibility.^39 70^ We invite further research to investigate how particular facets of the Filipino setting, including household characteristic and the role of public vaccination discourses in recent history, influence vaccination bargaining as compared to other contexts.

We are not aware of extensive data on varying vaccination profiles between children of the same household in the Philippines, but available data suggest a high number of vaccination dropouts, or children who did not receive subsequent vaccination doses after the first, in the country (including, eg, an estimated 30% dropout rate between the first and second dose of measles vaccine).^29^ Considering our finding that variations in the distribution of intra-household bargaining power over time can result in differing vaccination profiles, we suggest researchers and policymakers to consider bargaining processes in addition to other, more structural challenges for vaccination uptake when investigating approaches to ensure vaccination schedule adherence. Furthermore, future research could shed light on differences in vaccination patterns across children within the same household as a means to understand the variability in household vaccination decision-making.

This study has limitations. First, household bargaining inductively emerged during data collection and analysis, therefore we did not always actively or evenly probe on bargaining power and intra-household decision-making in early interviews. Because topics such as household finances can be difficult to discuss^23^ and might therefore not emerge evenly across interviews, we encourage future research, including research designed a priori to investigate intra-household dynamics. Second, vaccinating children might be perceived as a socially desirable action in our setting; respondents therefore could have felt compelled to overemphasise household dynamics that did not allow them to vaccinate their children despite wanting to do so. Third, a majority of our respondents were mothers. While this reflects how mothers are most often primary caregivers to small children in the Philippines, our data derived from other household members (such as fathers and grandparents) might have missed nuances from those members. We encourage future research that specifically aims at giving voice to household members currently underrepresented in this specific discourse, and that investigates the complex dynamics underlying preferences and bargaining approaches of different groups of household members. Finally, the purpose of this study was to tease out sources of bargaining power household members drew on in the vaccination decision-making process, and not to draw definitive links between a given source of power and positive or negative vaccination attitudes. We invite future research to investigate whether specific sources of power are more likely to lead to an uptake or rejection of childhood vaccination.

## Conclusion

To the best of our knowledge, this study is among the first to move beyond gendered imbalances when investigating the dynamics that shape the bargaining of childhood vaccination uptake. Considering the relevance of VH for global health, a better understanding of the circumstances in which decisions about vaccination are made, and how to address actors involved in these decisions, can guide strategising of vaccine confidence interventions.

Given the gendered dynamics of decision-making, which often limit mothers, we encourage the following: Target not only mothers in vaccination campaigns but also other household members who have more gendered power in the respective setting. Leverage the high relevance of physical presence by timing accessible vaccination offers accordingly, especially in cases where authoritative household members oppose vaccination. Inspire confidence in the own caregiving experience among household members favoring vaccination, and invoke interest and conviction about the topic, for example via nudging interventions,^71^ among household members with considerable bargaining power. Considering the observed diminished relevance of access to household finances in the context of vaccination but concerns that mothers’ relative bargaining power decreases as costs for vaccination uptake increase, programme planners should also continue to ensure that financial costs of accessing vaccination (including fees, transportation, loss of income, etc) remain low.

We invite future research that explores these potential avenues and further refines and tests our framework. We particularly encourage investigations of its applicability in other settings, such as contexts with differing household structures or socioeconomic backgrounds, and quantitative approaches that could further tease out patterns and provide nationally representative insights. We also encourage researchers to broaden the scope of the vaccination bargaining literature, including in high-income countries where bargaining patterns may vary, or by investigating bargaining as it relates to adult vaccination and how this can influence childhood vaccination bargaining and vice versa. We welcome researchers, policymakers and implementers involved in vaccine promotion efforts to engage with our framework, test its applicability across contexts and modify it in terms of key actors or the weight, type and interrelatedness of building blocks.

## Data Availability

Data are available upon reasonable request. Data are not publicly available due to the sensitive and personal nature of qualitative data and the collected information. Data may be available on request to authors, with restrictions following ethical approval. Please contact the corresponding author.
