# Supplementary material for: Bargaining and gendered authority: a framework to understand household decision-making about childhood vaccines in the Philippines
Source: BMJ Glob Health. 2022 Sep 30;7(9):e009781. doi: 10.1136/bmjgh-2022-009781 (PMC9528616; doi:10.1136/bmjgh-2022-009781)
Supplement: Supplementary data [file bmjgh-2022-009781supp001.pdf]

Supplemental file S1:
COREQ (Consolidated criteria for Reporting Qualitative research) Checklist

Item No.	Topic and Guide Questions	Report
**Domain 1: Research team and reflexivity**		
Personal characteristics		
1	Interviewer/ facilitator<br>(*Which author/s conducted the interview or focus group?*)	VE, MFA, JLG, JL and TAB conducted all interviews *(see supplementary file S2 for more details).*
2	Credentials<br>(*What were the researcher's credentials? E.g. PhD, MD*)	Bachelor's degree (VE, MFA, JLG, JL, TAB); Master's degree (JW, MDCR); PhD (SAM)
3	Occupation<br>(*What was their occupation at the time of the study?*)	All interviewers were employed as researchers at the Research Institute for Tropical Medicine (RITM), the Philippines, at time of data collection.
4	Gender<br>(*Was the researcher male or female?*)	The research team included female, male and non-binary individuals (*see supplementary file S2 for more details*).
5	Experience and training<br>(*What experience or training did the researcher have?*)	Interviewers had 2 to 15 years of previous experience in conducting qualitative research and were trained for three days on data collection procedures and ethical considerations (*see methods chapter for further information*).
Relationship with participants		
6	Relationship established<br>(*Was a relationship established prior to study commencement?*)	There was no personal relationship between interviewers and participants prior to data collection. When first contacting eligible participants upon recommendation and introduction by local health workers, interviewers introduced themselves and answered any open questions prior to enrolling the participant and scheduling the interview (*see protocol article*[1] *for further information*).
7	Participant knowledge of the interviewer	Interviewers introduced themselves and the study to participants. This introduction also included their own affiliation with RITM, their overarching interest in the topic

[1] Reñosa MDC, et al. 2021. *How can human-centered design build a story-based video intervention that addresses vaccine hesitancy and bolsters vaccine confidence in the Philippines? A mixed method protocol for project SALUBONG.* BMJ Open 11:e046814. doi:10.1136/bmjopen-2020-046814

	(*What did the participants know about the researcher? e.g. personal goals, reasons for doing the research*)	at hand, as well as the reasons for and goals of conducting this research.
8	Interviewer characteristics<br>(*What characteristics were reported about the interviewer/facilitator? e.g. Bias, assumptions, reasons and interests in the research topic*)	Following the Dengvaxia controversy and the resulting plummeting vaccination rates, RITM as the research arm of the Philippines Department of Health (DOH) identified vaccine hesitancy as a research and policy priority. This, and personal interest, sparked researchers' engagement in this topic. Interviewers were all Filipino nationals, one originating from an area close to the study setting. While this also was integral to building rapport with participants and collecting in-depth information on their personal experiences, it might also have resulted in biases. As part of the systematic debriefings conducted throughout data collection, emerging concerns and risks of biases were repeatedly discussed (*see protocol paper and supplementary file S2 for further information*).
**Domain 2: Study design**		
Theoretical framework		
9	Methodological orientation and Theory<br>(*What methodological orientation was stated to underpin the study? e.g. grounded theory, discourse analysis, ethnography, phenomenology, content analysis*)	The overarching study was guided by a human-centred design approach. Data collection for this manuscript was guided by a framework analysis approach (*see methods chapter for further information on data analysis processes*).
Participant selection		
10	Sampling<br>(*How were participants selected? e.g. purposive, convenience, consecutive, snowball*)	Participants were purposefully selected based on the vaccination record of their child showing delay or refusal of at least one recommended childhood vaccine *(see methods chapter and the protocol paper for more details)*.
11	Method of approach<br>(*How were participants approached? e.g. face-to-face, telephone, mail, email*)	In light of the pandemic situation at the time of data collection, participants were initially contacted via phone by a member of the study team after having expressed interest following an in-person visit of their respective healthcare worker (*see protocol paper for more details*).
12	Sample size<br>(*How many participants were in the study?*)	This article is based on interviews with 45 individuals (*see methods chapter for more details*).

13	Non-participation<br><br>(*How many people refused to participate or dropped out? Reasons?*)	There was a total of 11 respondents who declined to participate after thorough discussion of the study procedures. Reasons included busy schedules (n=2), husbands or their family members not allowing participation (n=4) and others not giving any specific reasons (n=5). No dropouts were noted.
Setting		
14	Setting of data collection<br><br>(*Where was the data collected? e.g. home, clinic, workplace*)	Given the pandemic situation, interviews were conducted online via a platform of the participant's choosing (*see protocol paper for more information*). Participants also chose the exact location from where to join the online interview; most participants were at their homeplace at the time of interview.
15	Presence of non-participants<br><br>(*Was anyone else present besides the participants and researchers?*)	Participants were encouraged to join the online interview alone and from a space they felt comfortable in. Due to the online nature of interviews, we cannot be sure whether in some cases other individuals were present for parts of the interview (*see methods chapter for more information*).
16	Description of sample<br><br>(*What are the important characteristics of the sample? e.g. demographic data, date*)	Characteristics of the sample are reported at the beginning of the results chapter.
Data collection		
17	Interview guide<br><br>(*Were questions, prompts, guides provided by the authors? Was it pilot tested?*)	Interviews were conducted following a piloted and refined semi-structured interview guide, focusing on participant's knowledge of and experiences with childhood vaccines, their decision-making processes, and their feedback on the intervention prototype of the overarching study. Intra-household dynamics emerged as a salient topic during the first few interviews and where then probed on in detail during consecutive interviews (*see methods chapter and protocol paper for further information*).
18	Repeat interviews<br><br>(*Were repeat interviews carried out? If yes, how many?*)	No repeat interviews were carried out.
19	Audio/visual recording<br><br>(*Did the research use audio or visual recording to collect the data?*)	All interviews were audio- and/or video-recorded.

20	Field notes<br><br>(*Were field notes made during and/or after the interview or focus group?*)	Interviewers took field notes during and/or immediately after the interviews which served as a basis for discussion during systematic debriefings.
21	Duration<br><br>(*What was the duration of the interviews or focus group?*)	Duration of the interviews is reported at the beginning of the results chapter.
22	Data saturation<br><br>(*Was data saturation discussed?*)	Interviews were halted when data saturation was achieved (*for more information see methods chapter; for more details on sample size considerations see protocol paper*).
23	Transcripts returned<br><br>(*Were transcripts returned to participants for comment and/or correction?*)	Transcripts were not returned to participants.
**Domain 3: analysis and findings**		
Data analysis		
24	Number of data coders<br><br>(*How many data coders coded the data?*)	The codebook was developed collaboratively by JW, MDCR and SAM with support of all authors. JW then coded the entire dataset while having regular debriefings with MDCR and SAM.
25	Description of the coding tree<br><br>(*Did authors provide a description of the coding tree?*)	The codebook was developed inductively based on the familiarization of the interview and focused on emerging intra-household dynamics. This included codes for key stakeholders in the decision-making process (mothers, fathers, maternal grandparents/extended family, paternal grandparents/extended family, as well as e.g. healthcare providers and neighbours), household decision-making processes and their outcomes, as well as the types of bargaining power drawn upon by key stakeholders during this decision-making process (*see methods chapter for more information*).
26	Derivation of themes<br><br>(*Were themes identified in advance or derived from the data?*)	Themes emerged inductively during data systematic debriefings and the coding of interviews (*see methods chapter for more information*).
27	Software<br><br>(*What software, if applicable, was used to manage the data?*)	NVivo 12 (12.6.0., QSR International) was used for coding the data.

28	Participant checking<br>(*Did participants provide feedback on the findings?*)	So far, findings have not been discussed with participants. However, we are considering conducting a larger outreach activity for sharing our results with participants upon concluding the overarching study.
Reporting		
29	Quotations presented<br>(*Were participant quotations presented to illustrate the themes/findings? Was each quotation identified? e.g. participant number*)	We present verbatim quotes throughout the results section. We use participant type, number of children and rural/urban area of residence as identifiers.
30	Data and findings consistent<br>(*Was there consistency between the data presented and the findings?*)	We closely link our findings to our data throughout the results section.
31	Clarity of major themes<br>(*Were major themes clearly presented in the findings?*)	Major themes, as outlined in Figure 1, are elaborated upon throughout the results section.
32	Clarity of minor themes<br>(*Is there a description of diverse cases or discussion of minor themes?*)	Minor themes and specific cases are discussed throughout the results section, including the two case studies.

Developed based on: Tong A, Sainsbury P, Craig J. Consolidated criteria for reporting qualitative research (COREQ): a 32-item checklist for interviews and focus groups. International Journal for Quality in Health Care. 2007. Volume 19, Number 6: pp. 349 – 357

Once you have completed this checklist, please save a copy and upload it as part of your submission. DO NOT include this checklist as part of the main manuscript document. It must be uploaded as a separate file.
