# Supplementary material for: Bargaining and gendered authority: a framework to understand household decision-making about childhood vaccines in the Philippines
Source: BMJ Glob Health. 2022 Sep 30;7(9):e009781. doi: 10.1136/bmjgh-2022-009781 (PMC9528616; doi:10.1136/bmjgh-2022-009781)
Supplement: Supplementary data [file bmjgh-2022-009781supp002.pdf]

Supplemental file S2:
Author Reflexivity Statement

1. **How does this study address local research and policy priorities?**

This study was designed in close partnership between researchers from the Heidelberg Institute of Global Health (HIGH), Germany, and the Research Institute for Tropical Medicine (RITM), the Philippines. Following the Dengvaxia controversy and the resulting plummeting vaccination rates, RITM as the research arm of the Philippines Department of Health (DOH) identified vaccine hesitancy as a research and policy priority. Further, this study aligns with the overarching priorities of the DOH, which is working to regain the public's trust and confidence in vaccination and health programs more broadly.

2. **How were local researchers involved in study design?**

The Dengvaxia controversy and its fallout became a major topic in the Philippines, in courses related to global health, and in regular conversations between MDCR, SAM and the broader team throughout and following MDCR's year of master's study where SAM served as their advisor. In this sense, the study design reflects a constant exchange between local and external researchers. Study procedures followed best practice approaches used at RITM for collecting and managing data in the Philippines. In light of COVID-associated restrictions, local researchers identified the need for and led the development of novel approaches to online data collection; decisions regarding adaptation of the study design were made collectively by the entire study team.

3. **How has funding been used to support the local research team?**

The larger research project has been used to leverage approximately 100,000 USD, approximately 45% of which were spent at the discretion of RITM partners. All decisions on funding allocation were made in partnership between MDCR and SAM. A majority of funds were used for data collection and local design of intervention material. Researchers (local and external) also drew from funder support for open-access publication. Additionally, this project has assisted local researchers to leverage additional funding to support their own projects and professional development, including a PhD grant for MDCR (approx. 40,000 USD) and funding for further research in this field (TAB, approx. 1,000 USD).

4. **How are research staff who conducted data collection acknowledged?**

All researchers involved in the collection of data have co-authored this manuscript.

5. **Do all members of the research partnership have access to study data?**

All members of the partnership have access to data.

6. **How was data used to develop analytical skills within the partnership?**

Local partners were involved in data analysis processes, starting with the systematic debriefings during data collection. Local researchers leading their own publications were supported by international partners throughout data analysis and writeup, building analytical capacity. This partnership is ongoing, with several capacity building activities for quantitative and qualitative analysis, as well as academic writing currently underway or scheduled.

7. **How have research partners collaborated in interpreting study data?**

Data interpretation commenced with routine, in-depth systematic debriefings which involved the entire study team, including local research partners. The topic of household bargaining emerged as highly salient during these debriefings, and a collective decision was made to further explore this topic. After data collection had concluded, JW initiated systematic analysis, regularly discussing the refined codebook with local (MDCR) and international (SAM) team members. Co-authors provided critical feedback on the manuscript across several versions to ensure contextualized interpretation of study data.

8. How were research partners supported to develop writing skills?

Throughout the collaboration, SAM, who has additional training as a journalist, provided scientific writing guidance to all team members. Four local early career research team members have already published their first first-authored publication on data originating from this study.

9. How will research products be shared to address local needs?

This article will be published open access. Additionally, we are repeatedly sharing our results in the form of policy briefs with the local policymaking bodies; results from this article will be included in subsequent policy briefs. Further, we have routinely presented our findings to RITM and DOH in several forums and meetings.

10. How is the leadership, contribution and ownership of this work by LMIC researchers recognised within the authorship?

Besides JW and SAM, all co-authors of this article are LMIC researchers. We acknowledge that JW and SAM are first and last author of this publication. This article is being led by JW as part of his PhD work, in which SAM serves as the supervisor. The decision on who would lead this article (as first, second and senior authors) was made collectively by the full research team. All local researchers involved in the data collection, analysis, and/or writeup are included as co-authors.

11. **How have early career researchers across the partnership been included within the authorship team?**

All co-authors of this manuscript are early career researchers (Defined as having less than 8 years of experience following completion of postgraduate research training). JW and MDCR are current doctoral students, and VE, JL and TAB are in the planning stages for pursuing graduate training in Global Health.

12. How has gender balance been addressed within the authorship?

Authors include non-binary, male, and female persons, and given the context of this work we repeatedly reflected on notions of gender and gender roles throughout data analysis and writeup. However, we do acknowledge that most co-authors identify as female, resulting in a formal gender imbalance in our list of co-authors. We sought to mitigate this gender imbalance by encouraging open conversation and honest reflection during routine systematic debriefings and manuscript writing. Constant reassurances and communication may have alleviated this gender imbalance.

13. How has the project contributed to training of LMIC researchers?

As highlighted above, all authors besides SAM are pre-doctoral researchers. In-depth training on data collection, analysis and writeup has been and/or will be conducted for LMIC-

based research team members, and several research team members have first-authored publications based on this project or are in the process of preparing such publications.

14. How has the project contributed to improvements in local infrastructure?

This project has not directly contributed to improvements in local infrastructure.

15. What safeguarding procedures were used to protect local study participants and researchers?

Primary data collection followed the tenets of the declaration of Helsinki and the Belmont report, as well as the Philippines' National Ethical Guidelines for Health and Health Related Research to protect study participants. With regard to the ongoing COVID-19 pandemic, the decision was made to shift all data collection online to minimize infection risk for both study participants and the research team. As part of regular systematic debriefings throughout data collection, concerns emerging for the local research team were addressed. In compliance with Philippines Republic Act 10173 or the Data Privacy Act of the 2012, all data was pseudonymized immediately after data collection to protect the identity of participants.
